# Poly[aqua­(μ_2_-oxalato)(4-oxidopyri­din­ium)erbium(II)]

**DOI:** 10.1107/S1600536808009380

**Published:** 2008-04-10

**Authors:** Chang-Sheng Gu, Xiao-Min Hao, Wen-Dong Song, Hai-Sheng Lin, De-Yun Ma

**Affiliations:** a College of Science, Guang Dong Ocean University, Zhanjiang 524088, People’s Republic of China; b College of Science, Guang Dong Ocean University, Zhanjiang 524088, People’s Republic of China; c College of Chemistry, South China University of Technology, Guangzhou 510640, People’s Republic of China

## Abstract

The title complex, [Er(C_5_H_5_NO)(C_2_O_4_)(H_2_O)]_*n*_, is a new erbium polymer based on oxalate and 4-oxidopyridinium ligands. The Er^II^ center is coordinated by six O atoms from three oxalate ligands, one O atom from a 4-oxidopyridinium ligand and one water mol­ecule, and displays a distorted square-anti­prismatic coordination geometry. The oxalate ligands are both chelating and bridging, and link Er^II^ ions, forming Er–oxalate layers in which the attached water and 4-oxidopyridinium units point alternately up and down. A mirror plane passes through the Er atom, one C, the oxide O and two oxalate O atoms. The layers are assembled into a three-dimensional supra­molecular network *via* inter­molecular hydrogen bonding and π–π stacking inter­actions [centroid–centroid distances of 3.587 (2) Å between parallel pyridinium rings]. Both the water mol­ecule and the 4-oxidopyridinium ligand are disordered over two sites in a 1:1 ratio.

## Related literature

For related literature, see: Yaghi *et al.* (1998 ▶, 2003 ▶); Serre *et al.* (2004 ▶); James (2003 ▶).
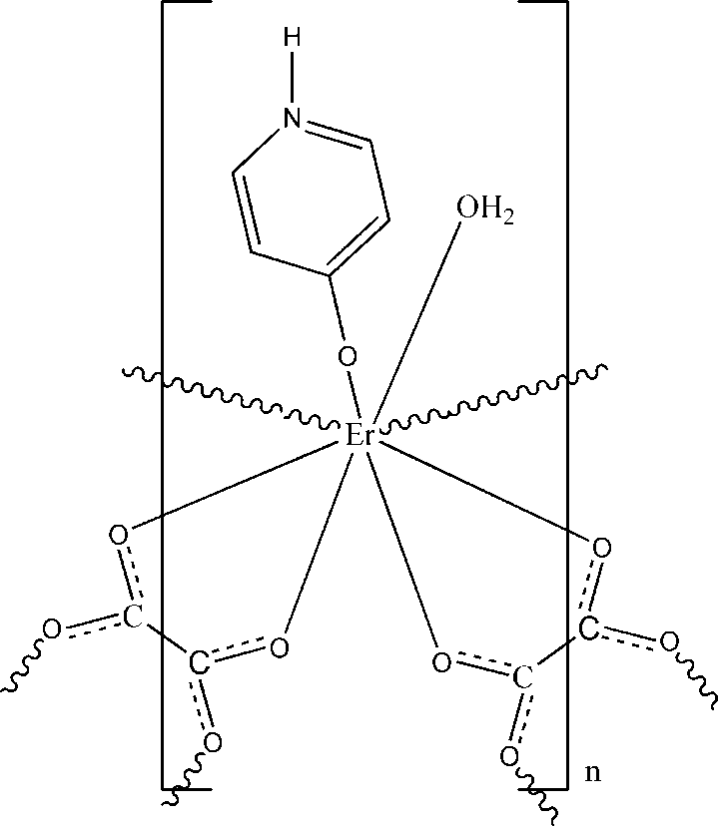


## Experimental

### 

#### Crystal data

[Er(C_5_H_5_NO)(C_2_O_4_)(H_2_O)]
*M* *_r_* = 412.41
Monoclinic, 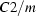

*a* = 16.8649 (2) Å
*b* = 11.1863 (2) Å
*c* = 6.5152 (1) Å
β = 112.213 (1)°
*V* = 1137.91 (3) Å^3^
*Z* = 4
Mo *K*α radiation
μ = 7.41 mm^−1^
*T* = 296 (2) K
0.21 × 0.19 × 0.13 mm

#### Data collection

Bruker APEXII area-detector diffractometer
Absorption correction: multi-scan (*SADABS*; Sheldrick, 1996 ▶) *T*_min_ = 0.241, *T*_max_ = 0.392
7274 measured reflections
1365 independent reflections
1341 reflections with *I* > 2σ(*I*)
*R*_int_ = 0.022

#### Refinement

*R*[*F* ^2^ > 2σ(*F* ^2^)] = 0.014
*wR*(*F* ^2^) = 0.036
*S* = 1.16
1365 reflections
105 parameters
39 restraints
H atoms treated by a mixture of independent and constrained refinement
Δρ_max_ = 0.53 e Å^−3^
Δρ_min_ = −0.88 e Å^−3^

### 

Data collection: *APEX2* (Bruker, 2004 ▶); cell refinement: *SAINT* (Bruker, 2004 ▶); data reduction: *SAINT*; program(s) used to solve structure: *SHELXS97* (Sheldrick, 2008 ▶); program(s) used to refine structure: *SHELXL97* (Sheldrick, 2008 ▶); molecular graphics: *SHELXTL* (Sheldrick, 2008 ▶); software used to prepare material for publication: *SHELXTL*.

## Supplementary Material

Crystal structure: contains datablocks I, global. DOI: 10.1107/S1600536808009380/zl2097sup1.cif

Structure factors: contains datablocks I. DOI: 10.1107/S1600536808009380/zl2097Isup2.hkl

Additional supplementary materials:  crystallographic information; 3D view; checkCIF report

## Figures and Tables

**Table 1:** Hydrogen-bond geometry (Å, °)

*D*—H⋯*A*	*D*—H	H⋯*A*	*D*⋯*A*	*D*—H⋯*A*
O1*W*—H2*W*⋯O1^i^	0.818 (10)	2.03 (4)	2.769 (4)	149 (8)
O1*W*—H1*W*⋯O2^i^	0.818 (10)	2.18 (6)	2.729 (4)	124 (6)
N1—H6⋯O4^ii^	0.86	2.41	3.041 (3)	130
N1—H6⋯O4^iii^	0.86	2.02	2.794 (3)	150

Symmetry codes: (i) 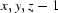; (ii) 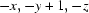; (iii) 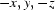.

**Fig. 1.**
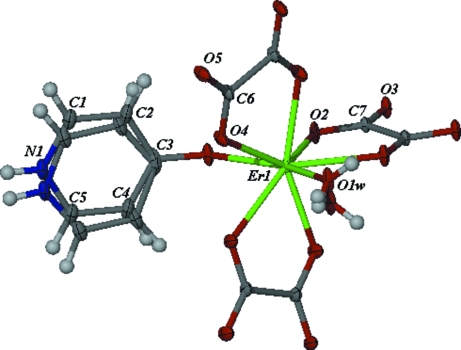
The structure of the title compound, showing the atom-numbering scheme and displacement ellipsoids drawn at the 30% probability level.

**Fig. 2.**
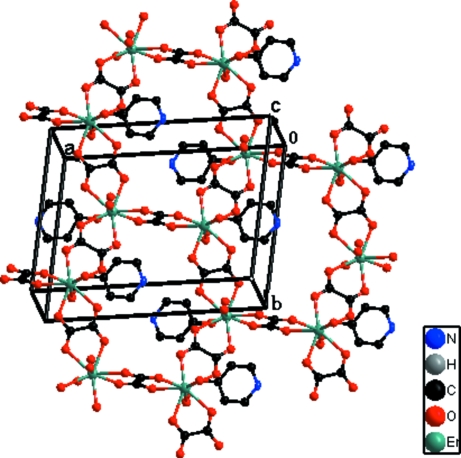
A layer view of (I). Hydrogen bonds are depicted as broken lines.

## supplementary crystallographic information

### Comment

The use of multifunctional organic linker molecules to polymerize metal centers into open-framework materials has led to the development of a rich field of chemistry (Yaghi *et al.*, 1998, 2003; Serre *et al.*, 2004; James, 2003) owing to the potential applications of these materials in catalysis, separation, gas storage and molecular recognition. Among such novel open-framework materials, lanthanide oxalates are particularly noteworthy. The wide variety of coordination modes of the oxalate anion permits the use of metal-oxalate units as excellent building blocks to construct a great diversity of frameworks ranging from discrete oligomeric entities to one-, two- and three-dimensional networks. Recently, we obtained the title erbium polymer (I), and its crystal structure is reported here.

The Er^II^ centre in the title compound exhibits a distorted square-antiprismatic coordination geometry, defined by six O atoms from three oxalate ligands, one O atom from the 4-oxidopyridinium ligand and one water molecule (Fig. 1). The oxalate ligands exhibit bidentate O atoms and link to the Er^II^ ions in a bridging mode to adjacent metal centres with Er—Er distances of 6.153 (2) Å and 6.112 (3) Å, respectively, thus forming Er-oxalate layers with the attached water and the 4-oxidopyridinium units that are alternatingly pointing up and down (Fig. 2). The layers are assembled into a three-dimensional supramolecular network *via* intermolecular O—H···O and N—H···O hydrogen bonding interactions (Table 1) involving the coordinated water molecules, N-protonated 4-hydroxypyridine, the hydroxy O atoms and the oxalate O atoms. They are also stabilized by π-π stacking interactions with centroid to centroid distances of 3.587 (2)A% between parallel pyridinium rings of neighboring complexes (at -*x*, *y*, 1 -*z*). The coordinated water molecule and the 4-oxidopyridinium ligands are located close to a mirror plane perpendicular to the b-axis of the unit cell and are disordered across this plane in a one to one ratio. As stated above the water molecules are engaged in hydrogen bonding to the hydroxide O atom O1 and to oxalate atom O2 (Table 1), and the orientation of the hydrogen O—H···O bond is equivalent but opposite for the two different disordered moieties, thus causing the disorder observed for the water molecule. The hydrogen bond formed by the 4-oxidopyridinium moiety is directed to either of the two symmetry equivalent oxalate oxygen atoms O4 (Table 1), and formation of either of the two H bonds is again responsible for the presence of the disorder observed.

### Experimental

A mixture of Er_2_O_3_ (0.5 mmol), oxalic acid (1 mmol), 4-hydroxypyridine (1 mmol) and H_2_O (10 ml) was placed in a 23 ml Teflon reactor, which was heated to 433 K for three days and then cooled to room temperature at a rate of 10 K h^-1^. The crystals obtained were washed with water and dryed in air.

### Refinement

In the initial refinement with disorder not taken into account both the water molecule and the 4-oxidopyridinium moiety showed significantly elongated thermal ellipsoids indicating disorder, and they were thus refined as being disordered over two positions across a crystallographic mirror plane perpendicular to the b-axis. The ADPs of the disordered atoms were restrained to be close to isotropic and those of equivalent atoms were set to be identical. Carbon-bound H atoms were placed in calculated positions and were treated as riding on the parent C atoms with C—H = 0.93 Å, N—H = 0.86 Å and with *U*_iso_(H) = 1.2 *U*_eq_(C, N); Water H atoms were tentatively located in difference Fourier maps and were refined with distance restraints of O–H = 0.85 Å and H···H = 1.39 Å, each within a standard deviation of 0.01 Å, and with *U*_iso_(H) = 1.5 *U*_eq_(O).

### Crystal data

**Table:**

[Er(C_5_H_5_NO)(C_2_O_4_)(H_2_O)]	*F*_000_ = 776
*M**_r_* = 412.41	*D*_x_ = 2.407 Mg m^−^^3^
Monoclinic, *C*2/*m*	Mo *K*α radiation λ = 0.71073 Å
Hall symbol: -C 2y	Cell parameters from 8000 reflections
*a* = 16.8649 (2) Å	θ = 1.7–26.0º
*b* = 11.1863 (2) Å	µ = 7.41 mm^−^^1^
*c* = 6.51520 (10) Å	*T* = 296 (2) K
β = 112.2130 (10)º	Block, white
*V* = 1137.91 (3) Å^3^	0.21 × 0.19 × 0.13 mm
*Z* = 4	

### Data collection

**Table:**

Bruker APEXII area-detector diffractometer	1365 independent reflections
Radiation source: fine-focus sealed tube	1341 reflections with *I* > 2σ(*I*)
Monochromator: graphite	*R*_int_ = 0.022
*T* = 296(2) K	θ_max_ = 27.5º
φ and ω scans	θ_min_ = 2.2º
Absorption correction: multi-scan(SADABS; Sheldrick, 1996)	*h* = −21→18
*T*_min_ = 0.241, *T*_max_ = 0.392	*k* = −14→14
7274 measured reflections	*l* = −8→8

### Refinement

**Table:**

Refinement on *F*^2^	Secondary atom site location: difference Fourier map
Least-squares matrix: full	Hydrogen site location: inferred from neighbouring sites
*R*[*F*^2^ > 2σ(*F*^2^)] = 0.014	H atoms treated by a mixture of independent and constrained refinement
*wR*(*F*^2^) = 0.036	*w* = 1/[σ^2^(*F*_o_^2^) + (0.019*P*)^2^ + 1.5595*P*] where *P* = (*F*_o_^2^ + 2*F*_c_^2^)/3
*S* = 1.16	(Δ/σ)_max_ = 0.001
1365 reflections	Δρ_max_ = 0.53 e Å^−^^3^
105 parameters	Δρ_min_ = −0.87 e Å^−^^3^
39 restraints	Extinction correction: none
Primary atom site location: structure-invariant direct methods	

### Special details

**Table:**

Geometry. All e.s.d.'s (except the e.s.d. in the dihedral angle between two l.s. planes) are estimated using the full covariance matrix. The cell e.s.d.'s are taken into account individually in the estimation of e.s.d.'s in distances, angles and torsion angles; correlations between e.s.d.'s in cell parameters are only used when they are defined by crystal symmetry. An approximate (isotropic) treatment of cell e.s.d.'s is used for estimating e.s.d.'s involving l.s. planes.
Refinement. Refinement of *F*^2^ against ALL reflections. The weighted *R*-factor *wR* and goodness of fit *S* are based on *F*^2^, conventional *R*-factors *R* are based on *F*, with *F* set to zero for negative *F*^2^. The threshold expression of *F*^2^ > σ(*F*^2^) is used only for calculating *R*-factors(gt) *etc*. and is not relevant to the choice of reflections for refinement. *R*-factors based on *F*^2^ are statistically about twice as large as those based on *F*, and *R*- factors based on ALL data will be even larger.

### Fractional atomic coordinates and isotropic or equivalent isotropic displacement parameters (Å^2^)

**Table:**

	*x*	*y*	*z*	*U*_iso_*/*U*_eq_	Occ. (<1)
N1	−0.02721 (14)	0.4755 (4)	0.2392 (5)	0.0325 (18)	0.50
H6	−0.0815	0.4701	0.2056	0.039*	0.50
C1	0.0218 (3)	0.3724 (4)	0.2667 (9)	0.0358 (13)	0.50
H1	−0.0041	0.2977	0.2487	0.043*	0.50
C2	0.1096 (3)	0.3811 (8)	0.3210 (9)	0.0381 (10)	0.50
H2	0.1425	0.3121	0.3394	0.046*	0.50
C3	0.14844 (16)	0.4927 (10)	0.3478 (5)	0.0326 (13)	0.50
C4	0.0994 (4)	0.5958 (8)	0.3203 (9)	0.0381 (10)	0.50
H4	0.1254	0.6705	0.3383	0.046*	0.50
C5	0.0116 (4)	0.5871 (4)	0.2660 (9)	0.0358 (13)	0.50
H5	−0.0212	0.6561	0.2476	0.043*	0.50
O1W	0.3241 (2)	0.4753 (8)	−0.1619 (6)	0.040 (3)	0.50
H1W	0.358 (4)	0.438 (6)	−0.200 (10)	0.060*	0.50
H2W	0.291 (4)	0.506 (8)	−0.276 (7)	0.060*	0.50
C6	0.20541 (15)	0.2749 (2)	−0.0746 (4)	0.0252 (5)	
C7	0.4856 (2)	0.5000	0.5987 (5)	0.0212 (6)	
Er1	0.311393 (8)	0.5000	0.17883 (2)	0.01752 (6)	
O1	0.23010 (16)	0.5000	0.3883 (4)	0.0347 (6)	
O2	0.40635 (16)	0.5000	0.5514 (4)	0.0313 (6)	
O3	0.54305 (16)	0.5000	0.7871 (4)	0.0324 (6)	
O4	0.19303 (11)	0.38280 (16)	−0.0507 (3)	0.0313 (4)	
O5	0.15399 (12)	0.20337 (17)	−0.2053 (3)	0.0338 (4)	

### Atomic displacement parameters (Å^2^)

**Table:**

	*U*^11^	*U*^22^	*U*^33^	*U*^12^	*U*^13^	*U*^23^
N1	0.0174 (18)	0.056 (6)	0.0245 (17)	−0.0017 (19)	0.0082 (14)	−0.0025 (18)
C1	0.0264 (18)	0.052 (4)	0.0305 (15)	−0.0077 (17)	0.0121 (13)	−0.0001 (17)
C2	0.0235 (17)	0.059 (3)	0.0322 (15)	−0.011 (2)	0.0107 (13)	0.005 (2)
C3	0.0204 (19)	0.062 (4)	0.0147 (15)	0.008 (5)	0.0055 (14)	−0.008 (5)
C4	0.0235 (17)	0.059 (3)	0.0322 (15)	−0.011 (2)	0.0107 (13)	0.005 (2)
C5	0.0264 (18)	0.052 (4)	0.0305 (15)	−0.0077 (17)	0.0121 (13)	−0.0001 (17)
O1W	0.0155 (15)	0.089 (9)	0.0155 (13)	0.001 (2)	0.0049 (11)	0.002 (2)
C6	0.0150 (12)	0.0301 (11)	0.0257 (12)	0.0024 (9)	0.0021 (10)	−0.0033 (9)
C7	0.0120 (15)	0.0322 (16)	0.0177 (14)	0.000	0.0036 (12)	0.000
Er1	0.00977 (9)	0.02597 (9)	0.01457 (8)	0.000	0.00206 (6)	0.000
O1	0.0134 (12)	0.0683 (19)	0.0206 (12)	0.000	0.0044 (10)	0.000
O2	0.0111 (11)	0.0639 (18)	0.0177 (11)	0.000	0.0041 (9)	0.000
O3	0.0122 (12)	0.0645 (18)	0.0186 (12)	0.000	0.0038 (10)	0.000
O4	0.0161 (9)	0.0284 (8)	0.0392 (10)	0.0039 (7)	−0.0011 (8)	−0.0073 (7)
O5	0.0187 (9)	0.0307 (9)	0.0374 (10)	0.0035 (8)	−0.0058 (7)	−0.0064 (8)

### Geometric parameters (Å, °)

**Table:**

N1—C1	1.3900	C6—O5	1.249 (3)
N1—C5	1.3900	C6—C6^i^	1.553 (5)
N1—H6	0.8600	C7—O3	1.243 (4)
C1—C2	1.3900	C7—O2	1.253 (4)
C1—H1	0.9300	C7—C7^ii^	1.537 (6)
C2—C3	1.3900	Er1—O1	2.271 (3)
C2—H2	0.9300	Er1—O1W^iii^	2.326 (3)
C3—O1	1.303 (3)	Er1—O5^i^	2.3388 (19)
C3—C4	1.3900	Er1—O5^iv^	2.3388 (19)
C4—C5	1.3900	Er1—O2	2.349 (2)
C4—H4	0.9300	Er1—O3^ii^	2.380 (2)
C5—H5	0.9300	Er1—O4	2.3839 (17)
O1W—Er1	2.326 (3)	Er1—O4^iii^	2.3839 (17)
O1W—H1W	0.818 (10)	O1—C3^iii^	1.303 (4)
O1W—H2W	0.818 (10)	O3—Er1^ii^	2.380 (2)
C6—O4	1.245 (3)	O5—Er1^i^	2.3388 (19)
			
C1—N1—C5	120.0	O1W—Er1—O5^iv^	94.3 (2)
C1—N1—H6	120.0	O5^i^—Er1—O5^iv^	153.17 (9)
C5—N1—H6	120.0	O1—Er1—O2	73.12 (9)
C2—C1—N1	120.0	O1W^iii^—Er1—O2	135.51 (11)
C2—C1—H1	120.0	O1W—Er1—O2	135.51 (11)
N1—C1—H1	120.0	O5^i^—Er1—O2	82.54 (5)
C1—C2—C3	120.0	O5^iv^—Er1—O2	82.54 (5)
C1—C2—H2	120.0	O1—Er1—O3^ii^	141.24 (9)
C3—C2—H2	120.0	O1W^iii^—Er1—O3^ii^	67.96 (11)
O1—C3—C4	120.4 (7)	O1W—Er1—O3^ii^	67.96 (11)
O1—C3—C2	119.5 (7)	O5^i^—Er1—O3^ii^	76.89 (5)
C4—C3—C2	120.0	O5^iv^—Er1—O3^ii^	76.89 (5)
C5—C4—C3	120.0	O2—Er1—O3^ii^	68.12 (8)
C5—C4—H4	120.0	O1—Er1—O4	79.89 (7)
C3—C4—H4	120.0	O1W^iii^—Er1—O4	79.87 (16)
C4—C5—N1	120.0	O1W—Er1—O4	72.16 (15)
C4—C5—H5	120.0	O5^i^—Er1—O4	68.77 (6)
N1—C5—H5	120.0	O5^iv^—Er1—O4	135.04 (6)
Er1—O1W—H1W	133 (5)	O2—Er1—O4	136.92 (6)
Er1—O1W—H2W	123 (4)	O3^ii^—Er1—O4	130.40 (6)
H1W—O1W—H2W	104.2 (17)	O1—Er1—O4^iii^	79.89 (7)
O4—C6—O5	126.9 (2)	O1W^iii^—Er1—O4^iii^	72.16 (15)
O4—C6—C6^i^	116.0 (3)	O1W—Er1—O4^iii^	79.87 (16)
O5—C6—C6^i^	117.1 (3)	O5^i^—Er1—O4^iii^	135.04 (6)
O3—C7—O2	127.1 (3)	O5^iv^—Er1—O4^iii^	68.77 (6)
O3—C7—C7^ii^	116.8 (4)	O2—Er1—O4^iii^	136.92 (6)
O2—C7—C7^ii^	116.0 (4)	O3^ii^—Er1—O4^iii^	130.40 (6)
O1—Er1—O1W^iii^	150.24 (12)	O4—Er1—O4^iii^	66.72 (8)
O1—Er1—O1W	150.24 (12)	C3—O1—Er1	135.2 (2)
O1W^iii^—Er1—O1W	13.6 (5)	C3^iii^—O1—Er1	135.2 (2)
O1—Er1—O5^i^	98.41 (5)	C7—O2—Er1	120.1 (2)
O1W^iii^—Er1—O5^i^	94.3 (2)	C7—O3—Er1^ii^	118.9 (2)
O1W—Er1—O5^i^	81.0 (2)	C6—O4—Er1	118.12 (15)
O1—Er1—O5^iv^	98.41 (5)	C6—O5—Er1^i^	118.91 (16)
O1W^iii^—Er1—O5^iv^	81.0 (2)		

Symmetry codes: (i) −*x*+1/2, −*y*+1/2, −*z*; (ii) −*x*+1, −*y*+1, −*z*+1; (iii) *x*, −*y*+1, *z*; (iv) −*x*+1/2, *y*+1/2, −*z*.

### Hydrogen-bond geometry (Å, °)

**Table:**

*D*—H···*A*	*D*—H	H···*A*	*D*···*A*	*D*—H···*A*
O1W—H2W···O1^v^	0.818 (10)	2.03 (4)	2.769 (4)	149 (8)
O1W—H1W···O2^v^	0.818 (10)	2.18 (6)	2.729 (4)	124 (6)
N1—H6···O4^vi^	0.86	2.41	3.041 (3)	130
N1—H6···O4^vii^	0.86	2.02	2.794 (3)	150

Symmetry codes: (v) *x*, *y*, *z*−1; (vi) −*x*, −*y*+1, −*z*; (vii) −*x*, *y*, −*z*.
