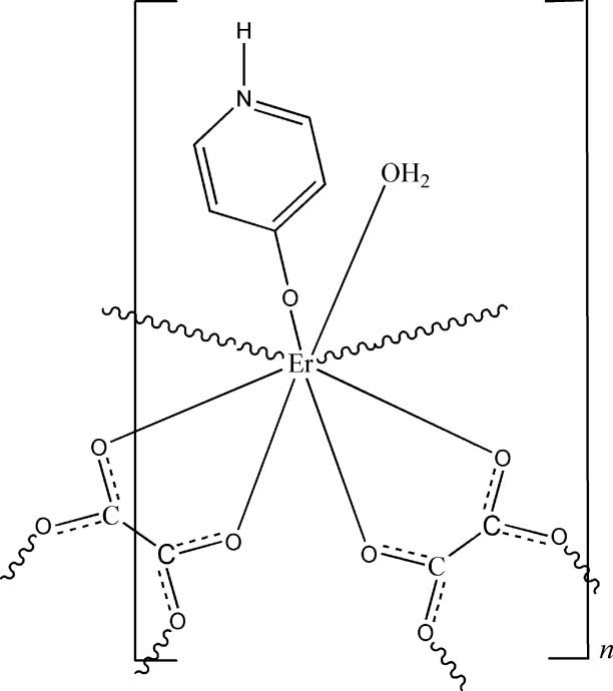# Poly[aqua­(μ_2_-oxalato)(4-oxidopyri­din­ium)erbium(II)]. Corrigendum

**DOI:** 10.1107/S1600536808037392

**Published:** 2008-11-13

**Authors:** Chang-Sheng Gu, Xiao-Min Hao, Wen-Dong Song, Hai-Sheng Lin, De-Yun Ma

**Affiliations:** a College of Science, Guang Dong Ocean University, Zhanjiang 524088, People’s Republic of China; b College of Science, Guang Dong Ocean University, Zhanjiang 524088, People’s Republic of China; c College of Chemistry, South China University of Technology, Guangzhou 510640, People’s Republic of China

## Abstract

The chemical name in the title, the formula and the scheme of the paper by Gu, Hao, Song, Lin & Ma [*Acta Cryst.* (2008), E**64**, m649–m650] are corrected.

## Abstract 2

Corrigendum to *Acta Cryst.* (2008), E**64**, m649–m650.

In the paper by Gu, Hao, Song, Lin & Ma [*Acta Cryst.* (2008), E**64**, m649–m650], the chemical name in the title, the formula and the scheme are incorrect. The correct title should be ‘Poly[aqua­sesqui(μ_2_-oxalato)(4-oxidopyri­din­ium)erbium(III)]’, [Er(C_2_O_4_)_1.5_(C_5_H_5_NO)(H_2_O)]_*n*_, and the correct scheme is shown below. Note the revised oxidation state for erbium(III) which is given as erbium(II) twice in the original *Abstract*.